# Lack of evidence for a role of hydrophobins in conferring surface hydrophobicity to conidia and hyphae of *Botrytis cinerea*

**DOI:** 10.1186/1471-2180-11-10

**Published:** 2011-01-13

**Authors:** Andreas Mosbach, Michaela Leroch, Kurt W Mendgen, Matthias Hahn

**Affiliations:** 1 Department of Biology, University of Kaiserslautern, Gottlieb-Daimler-Straße, 67663 Kaiserslautern, Germany; 2 Department of Biology, University of Konstanz, Universitätsstraße 10, 78457 Konstanz, Germany

## Abstract

**Background:**

Hydrophobins are small, cysteine rich, surface active proteins secreted by filamentous fungi, forming hydrophobic layers on the walls of aerial mycelia and spores. Hydrophobin mutants in a variety of fungi have been described to show 'easily wettable' phenotypes, indicating that hydrophobins play a general role in conferring surface hydrophobicity to aerial hyphae and spores.

**Results:**

In the genome of the grey mould fungus *Botrytis cinerea*, genes encoding three hydrophobins and six hydrophobin-like proteins were identified. Expression analyses revealed low or no expression of these genes in conidia, while some of them showed increased or specific expression in other stages, such as sclerotia or fruiting bodies. Bhp1 belongs to the class I hydrophobins, whereas Bhp2 and Bhp3 are members of hydrophobin class II. Single, double and triple hydrophobin knock-out mutants were constructed by consecutively deleting *bhp1*, *bhp2 *and *bhp3*. In addition, a mutant in the hydrophobin-like gene *bhl1 *was generated. The mutants were tested for germination and growth under different conditions, formation of sclerotia, ability to penetrate and infect host tissue, and for spore and mycelium surface properties. Surprisingly, none of the *B. cinerea *hydrophobin mutants showed obvious phenotypic defects in any of these characters. Scanning electron microscopy of the hydrophobic conidial surfaces did not reveal evidence for the presence of typical hydrophobin 'rodlet' layers.

**Conclusions:**

These data provide evidence that in *B. cinerea*, hydrophobins are not involved in conferring surface hydrophobicity to conidia and aerial hyphae, and challenge their universal role in filamentous fungi. The function of some of these proteins in sclerotia and fruiting bodies remains to be investigated.

## Background

Filamentous fungi produce unique proteins called hydrophobins that are secreted and cover the walls of spores and hyphae with a hydrophobic layer [1]. Structurally, hydrophobins are characterised by their small size and the presence of eight cysteine residues which are arranged in a conserved array and form four pairs of disulphide bridges. By their ability to aggregate to amphipathic membranes, they attach to the surface of the hydrophilic fungal cell wall, thereby exposing the hydrophobic layer to the outside [2]. By scanning electron microscopy, hydrophobin layers can often be recognised by the formation of rodlets of characteristic dimensions [3]. Hydrophobin aggregates are highly resistant against treatments that are used for solubilising proteins. Based on their amino acid sequences, hydropathy profiles and solvent solubility, two classes of hydrophobins are distinguished. While class I hydrophobin aggregates are extremely stable, and can be dissociated only in trifluoroacetic acid and formic acid, class II hydrophobin aggregates can be solubilised in hot sodium dodecyl sulphate (SDS) or 60% ethanol [2].

Hydrophobins have been shown to serve several basic functions in fungi. By covering hyphal walls with a hydrophobic surface layer, they allow hyphae to escape from aqueous substrates and to develop aerial mycelia [1]. Similarly, conidia are often covered with rodlet layers, which facilitate their dispersal by air or water droplets. Loss of the hydrophobin layers by targeted mutagenesis of hydrophobin genes can lead to drastic reduction in surface hydrophobicity, resulting in 'easily wettable' phenotypes [2]. In the rice pathogen *Magnaporthe oryzae *mutants in the class I hydrophobin Mpg1 produced easily wettable conidia and hyphae lacking rodlets, and were defective in appressorium formation and host infection. This was attributed to the inability of the germ tubes to firmly attach to the hydrophobic plant cuticle and to appropriately sense surface features leading to appressorium differentiation [4,5]. In the same fungus, the class II hydrophobin Mhp1 was also found to be involved in hyphal surface hydrophobicity and for pathogenesis [6]. The tree pathogen *Ophiostoma ulmi *produces cerato-ulmin, a class II hydrophobin which is a wilt-inducing toxin. Regarding its role in pathogenesis, a final conclusion has not yet been reached. While toxin-deficient mutants were not affected in pathogenicity, their phenotypes indicated that it contributes to the fitness of the spores of *O. ulmi *[7,8]. Similarly, hydrophobin mutations in the tomato pathogen *Cladosporium fulvum *did not impair the mutant strains to cause disease [9].

*Botrytis cinerea *(teleomorph *Botryotinia fuckeliana*) is a necrotrophic plant pathogenic ascomycete with a wide host range, including economically important fruits, vegetables and ornamental flowers. After colonisation of the host tissue, the fungus forms aerial mycelia that produce large numbers of conidia, which are the main source of new infections. Due to their surface hydrophobicity, conidia adhere easily to the plant surface [10]. This initial adhesion is relatively weak and followed by stronger attachment immediately after emergence of the germ tube [11]. Germ tubes secrete an ensheathing film that appears to mediate adhesion to hydrophobic and hydrophilic substrates. The biochemical composition of the film has been analysed, and was found to consist mainly of carbohydrates and proteins, plus minor amounts of lipids [12]. Germination of *B. cinerea *conidia has been found to depend both on the availability of nutrients and on physical surface properties. In solutions containing sugars as sole organic nutrients, efficient germination occurs only on a hard surface. In the absence of nutrients, germination can still be induced on hard, hydrophobic surfaces [13]. Induction of germination by hard hydrophobic surfaces has also been described for conidia of other plant pathogenic fungi, namely *Colletotrichum graminicola *and *Phyllosticta ampelicida *[14,15]. These data indicate that the hydrophobic surface properties of conidia are a prerequisite for appropriate surface sensing under nutrient-limiting conditions.

In order to test the role of hydrophobins in conidial and hyphal hydrophobicity, and therefore possibly in hydrophobic surface sensing, we performed a systematic search for the presence of hydrophobin genes in the *B. cinerea *genome, analysed their expression, and performed a functional analysis of three hydrophobin genes and a hydrophobin-like gene. Surprisingly, mutants lacking all these genes were found to be phenotypically indistinguishable from the wild type in all parameters tested. Our results challenge the concept that hydrophobins are generally required for the formation of hydrophobic surface layers in conidia and hyphae of higher fungi.

## Results

### Cloning and sequence analysis of *Botrytis cinerea *hydrophobin genes

In the *B. cinerea *strain B05.10 genome sequence, three hydrophobin encoding genes were identified. Using *Magnaporthe oryzae *class I hydrophobin Mpg1 [4] as a query in a blastp search, a protein (BC1G_15273) with weak homology was detected. Its size, arrangement of the eight conserved cysteines, and overall hydropathicity was similar to *M. oryzae *Mpg1 and other class I hydrophobins, and it was called Bhp1 (for '*Botrytis *hydrophobin'). Using *M. oryzae *class II hydrophobin Mhp1 [6] in another blastp query, the *B. cinerea *proteins BC1G_03994 (called Bhp2) and BC1G_01012 (called Bhp3) were found to show significant homologies (E values < e^-10^). With blastp and tblastn searches using known hydrophobin proteins, no further hydrophobin genes were identified in the *B. cinerea *genome. The identification of hydrophobin encoding genes in fungal genomes is sometimes difficult due to their small size, the variable spacing between the cysteine encoding codons, and their low sequence homologies, in particular among class I hydrophobin genes. In order to identify further candidates for *B. cinerea *hydrophobins, a systematic search was performed in the published genome sequences of *B. cinerea *strains B05.10 and T4. The following search parameters were used: a) Total size of the protein smaller than 250 amino acids; b) Presence of at least 6 cysteines, four of them in a tandem arrangement separated by two further cysteine residues (full cysteine motive of hydrophobins: C-(X_n_)-CC-(X_n_)-C-(X_n_)-C-(X_n_)-CC-(X_n_)-C); c) Prediction of a signal peptide. The search resulted in the identification of six further hydrophobin-like *B. cinerea *proteins, which all had a small size (98-234 aa), and a similar pattern of eight cysteines after manual correction of annotations (Table 1; additional file 1: Table S1). Examination of their hydropathicity revealed that five of these proteins are significantly less hydrophobic within the cysteine-rich region when compared to confirmed class I and II hydrophobins (Table 1). Only the protein encoded by BC1G_01003 (called Bhl1, for '*Botrytis *hydrophobin-like'), showed a hydrophobicity similar to Bhp1. However, the cysteine spacing of Bhl1 differs somewhat from that of confirmed class I hydrophobins [16] (Table 1), it has a distinct hydropathy profile (additional file 2: Figure S1), and it lacks homology to other fungal hydrophobins (data not shown).

**Table 1:** Sequence characteristics of *B. cinerea *hydrophobins and hydrophobin-like proteins.

Name/predicted class	Size	Spacing of cysteine residues						GRAVY
Bhp1 (BC1G_15273)	111/93	**N- 34-C- 7 **	**-CC- 18 **	**-C- 15 **	**-C- 5 **	**-CC- 17 **	**-C- 7**	0.57

Consensus spacing class I		**N- Xn-C- (5-8)**	**-CC-(17-39)**	**-C-(8-23)**	**-C-(5-6)**	**-CC-(6-18)**	**-C-(2-13)**	

Bhp2 (BC1G_03994)	98/77	**N- 33-C- 6 **	**-CC- 11**	**-C- 16**	**-C- 8 **	**-CC- 10**	**-C- 6**	0.42

Bhp3 (BC1G_01012)	98/80	**N- 34-C- 8 **	**-CC- 11**	**-C- 16**	**-C- 8 **	**-CC- 10**	**-C- 3**	0.30

Consensus spacing class II		**N- Xn-C-(9-10)**	**-CC- 11**	**-C- 16**	**-C-(6-9)**	**-CC- 10**	**-C- (3-7)**	

Bhl1 (BC1G_01003)	145/125	**N- 60-C- 9 **	**-CC- 31 **	**-C- 8 **	**-C- 7 **	**-CC- 16 **	**-C- 6**	0.76

BC1G_02483	234/211	**N- 82-C- 8 **	**-CC- 7**	** -C- 5 **	**-C- 9 **	**-CC- 8 **	**-C- 107**	-0.10

BC1G_03277	178/160	**N-111-C- 7 **	**-CC- 10 **	**-C- 17 **	**-C- 8 **	**-CC- 12 **	**-C- 5**	-0.43

BC1G_04521	181/157	**N-120-C- 7 **	**-CC- 10 **	**-C- 10 **	**-C- 9 **	**-CC- 4 **	**-C- 13**	0.01

BC1G_11117	109/88	**N- 35-C- 10 **	**-CC- 15 **	**-C- 18 **	**-C- 8 **	**-CC- 11 **	**-C- 4**	-0.77

BC1G_12747	106/86	**N- 37-C- 3 **	**-CC- 10 **	**-C- 13 **	**-C- 18 **	**-CC- 4**	**-C- 13**	-0.28

For the three hydrophobins Bhp1 (class I), Bhp2 and Bhp3 (both class II), and for six hydrophobin-like proteins, the cysteine spacing is shown. Consensus cysteine spacings for class I and class II proteins were taken from [16]. The sizes (amino acids) of the unprocessed and processed proteins are indicated. N: N-terminus; Xn: Undefined number of amino acids; Underlined: Strictly conserved spacing; GRAVY: Grand average of hydropathicity of the region covering the eight cysteines. Positive GRAVY values indicate hydrophobicity [53].

**Figure 1:**
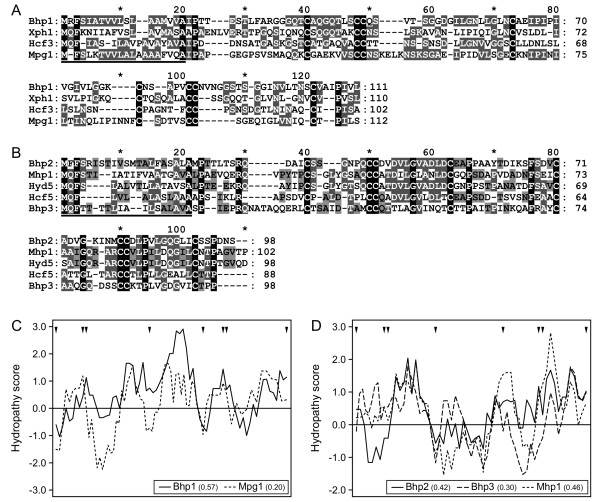
**Sequence alignments and hydropathy plots of *B. cinerea *hydrophobins and confirmed class I and II hydrophobins**. A: Amino acid alignment of Bhp1 and class I hydrophobins. B: Amino acid alignment of Bhp2/3 and class II hydrophobins. The signal peptides are underlined. Hcf3 (Acc.: CAD92803) and Hcf5 (Acc.: CAC27408) from *Cladosporium fulvum*; Hyd5 (Acc.: AAN76355) from *Fusarium verticillioides*; Mpg1 (Acc.: P52751) and Mhp1 (Acc.: AAD18059) from *M. oryzae*; Xph1 (Acc.: CAC43386) from *X. parietina*. C and D: Hydropathy plots with Bhp1 and *M. oryzae *Mpg1 (left), and with Bhp2, Bhp3 and *M. oryzae *Mhp1 (right). Hydropathy values were calculated for the sequences covering the eight cysteines (window size for calculation: 7 amino acids). Positive values indicate regions of high hydrophobicity. Positions of cysteine residues are marked by triangles. Grand average of hydropathicity (GRAVY) of the analysed region is indicated in parentheses.

Bhp1 is 111 amino acids long and contains eight cysteines with spacing as described for the class I hydrophobin consensus sequence [16]. It shows 30% identity to Xph1 of the lichen fungus *Xanthoria parietina*, and 29% identity to Mpg1 of *Magnaporthe oryzae *(Figure 1A). The hydropathy plot of Bhp1 shows similarity to that of Mpg1 and of other class I hydrophobins (Figure 1C; data not shown). Bhp2 and Bhp3 are both 98 amino acids long and 27% identical to each other. Both proteins match the consensus cysteine spacing of class II hydrophobins (Table 1) [16]. Bhp2 shares 37%, and Bhp3 29% identity with *M. oryzae *Mhp1 (Figure 1B). The hydropathy plots of Bhp2 and Mhp1 are similar (Figure 1D).

### Comparison of hydrophobin genes in *B. cinerea *and *Sclerotinia sclerotiorum*

A comparison of the genes that are encoding hydrophobins and hydrophobin-like proteins in the genomes of *B. cinerea *and the closely related *S. sclerotiorum *was performed (additional file 1: Table S1). For all except one (BC1G_12747) of the *B. cinerea *proteins, apparent orthologues were found in *S. sclerotiorum*. The proteins encoded by BC1G_11117 and SS1G_01003 are bidirectional best hits in blastp queries; however their overall sequence similarity (33% identity) is rather low.

### Expression of hydrophobin and hydrophobin-like genes during *B. cinerea *development

**Figure 2:**
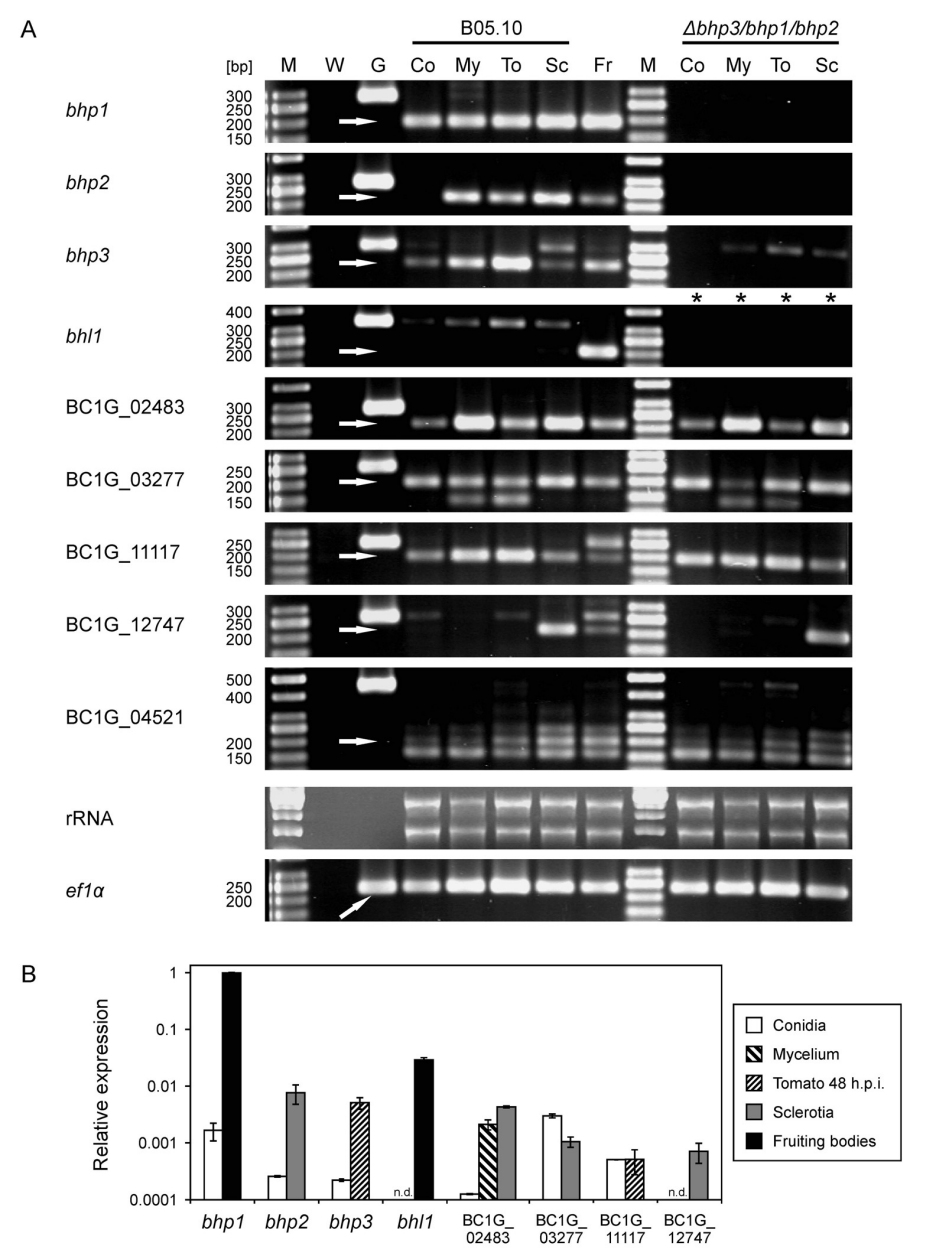
**Expression analysis of the hydrophobin genes *bhp1*, *bhp2 *and *bhp3*, and six hydrophobin-like genes**. A: Results of semi-quantitative RT-PCR, showing gene expression in different developmental stages of wild type B05.10, the hydrophobin triple mutant *Δbhp3/bhp1/bhp2*, and the *Δbhl1 *mutant (lanes with cDNA from *Δbhl1 *labelled with stars). M: Size markers, with relevant sizes indicated [bp]; W: Water control; G: Genomic DNA; Co: Resting conidia; My: mycelium (15 h.p.i.); To: Infected tomato leaves (48 h.p.i.); Sc: Sclerotia; Fr: Fruiting bodies. An EF1α encoding fragment was amplified as positive control. Arrows indicate positions of bands based on cDNA (in case of *ef1α*, the size of cDNA and genomic DNA is identical). Undiluted first-strand cDNA was amplified with 35 cycles, except for *ef1α *cDNA, which was amplified from 1:10 diluted first-strand cDNA. The multiple bands obtained with BC1G_04521-specific primers might be due to different splicing variants. The weak bands indicating the presence of wild type *bhp3 *genomic DNA in the triple hydrophobin mutant seem to result from the presence of few remaining, non-transformed nuclei. B: Results of real-time RT-PCR, showing gene expression in conidia and selected growth stages of strain B05.10, except for fruiting bodies which were from a cross of *B. cinerea *field isolates. Hydrophobin expression levels are shown relative to the mean of *actin *and *ef1α *expression.

To analyse the expression profiles of *bhp1*, *bhp2 *and *bhp3*, and the six hydrophobin-like genes, RNA from different developmental stages of *B. cinerea *was isolated and analysed by reverse transcription-PCR. As shown in Figure 2A, transcripts of *bhp1*, *bhp2 *and *bhp3*, as well as the *ef1α *gene which was used as positive control, could be detected in mycelia, infected tomato leaves 48 h.p.i. and mature sclerotia of the wild type strain B05.10, as well as in fruiting bodies from the cross of two *B. cinerea *field isolates. Except for *bhp2*, expression of all these genes was also visible in the conidial state. Generally, expression levels of the three hydrophobin genes appeared to be rather low. Transcripts of the hydrophobin-like genes BC1G_02483, BC1G_03277, BC1G_11117 and BC1G_04521 were also detected in all developmental stages tested, but with apparently variable expression levels. In contrast, expression of BC1G_12747 was largely restricted to sclerotia, and *bhl1 *transcripts were only observed in fruiting bodies. To estimate the expression levels of the genes more precisely, quantitative RT-PCR was performed (Figure 2B). For each of the genes, expression in conidia was compared to that in the stage(s) that appeared to show strongest expression. Expression of all genes in conidia was rather weak. Highest levels of expression were observed for *bhp1 *and *bhl1 *in fruiting bodies, in particular *bhp1 *reached expression levels similar to *actin *and *ef1α*. The increased expression of *bhp2*, BC1G_02483 and BC1G_12747 in sclerotia was also confirmed.

### Targeted deletion of *bhp1*, *bhp2*, *bhp3 *and *bhl1*

To analyse their functions, the hydrophobin genes *bhp1*, *bhp2 *and *bhp3 *were consecutively deleted. Hydrophobin single knock-out mutants were constructed by using hygromycin or nourseothricin cassettes for selection. For double knock-out mutants, both cassettes were sequentially used. Finally, for generating a triple knock-out mutant, a *Δbhp3/bhp1 *double mutant was transformed with a *bhp2 *knock-out construct carrying a phleomycin resistance cassette as a third selectable marker. Additionally, a knock-out mutant of the hydrophobin-like gene *bhl1 *was created. All transformants were verified by PCR analysis (data not shown), and by RT-PCR using cDNA from different developmental stages (Figure 2A). No transcripts of *bhp1*, *bhp2 *and *bhp3 *could be detected in the hydrophobin triple mutant in any of the growth stages tested. In the same way, no transcripts of genes that had been deleted could be amplified from hydrophobin double knock-out strains (additional file 3: Figure S2). The expression levels of the five hydrophobin-like genes BC1G_02483, BC1G_03277, BC1G_11117, BC1G_12747 and BC1G_04521 in the hydrophobin triple mutant appeared to be similar to the wild type, as far as this could be estimated from semi-quantitative RT-PCR. Because transcripts of *bhl1 *could be unambiguously detected only in fruiting bodies (Figure 2A), which were unavailable from *Δbhl1 *mutants, verification of the *Δbhl1 *strain by RT-PCR analysis was not possible.

### Growth, differentiation and infection behaviour of the hydrophobin mutants

**Figure 3:**
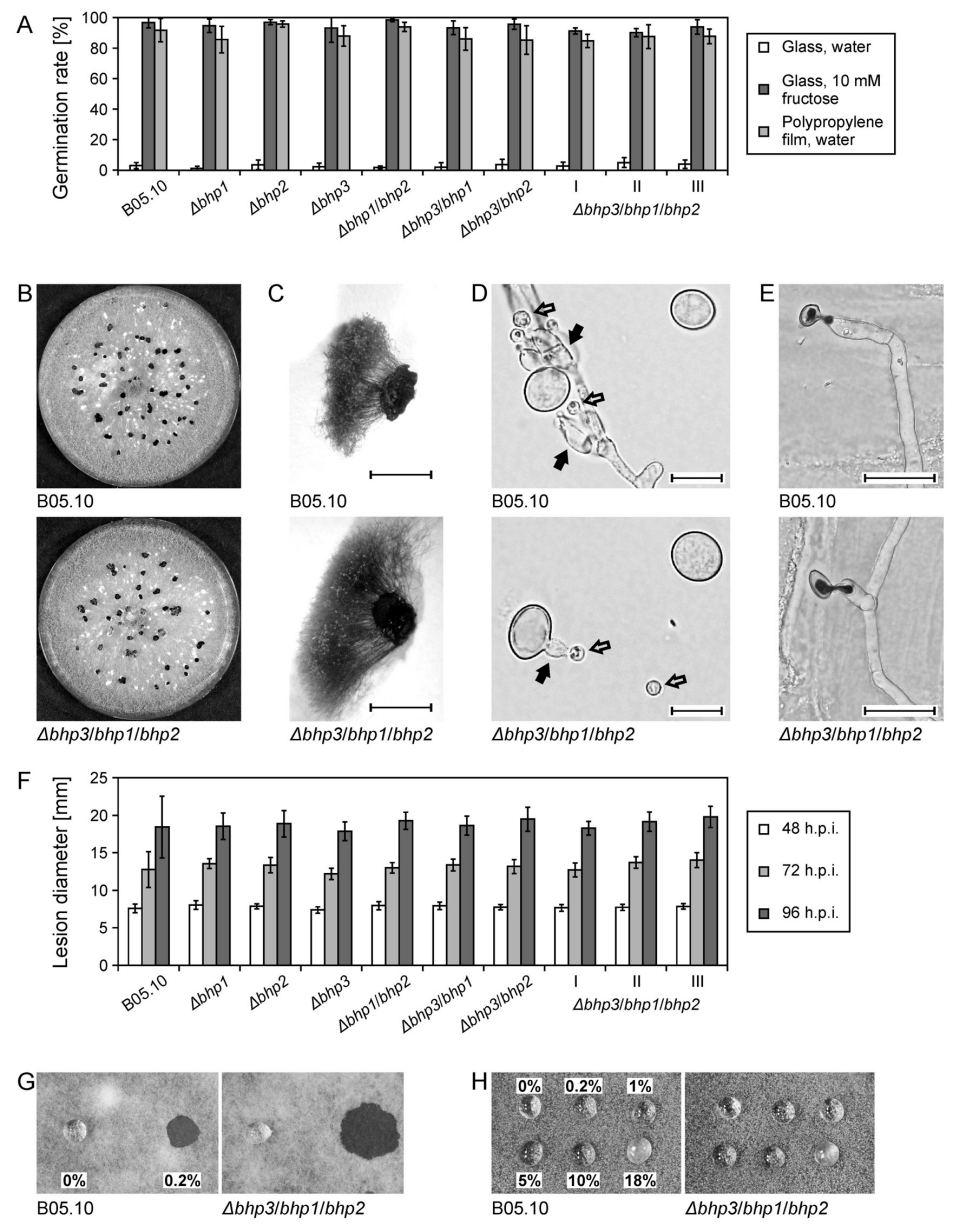
**Phenotypic characterisation of hydrophobin mutants**. A: Germination rates under different conditions, 24 h.p.i. I, II, II: Three transformants of hydrophobin triple mutant. Standard deviations are shown. B: Sclerotia formation on Gamborg agar plates. C: Germinated sclerotia with conidiophores and macroconidia (scale bar: 3 mm). D: Microconidia (hollow arrows) produced on phialides (filled arrows). Phialides were observed on branching hyphae and on macroconidia of B05.10 and the triple mutant (scale bar: 10 μm). E: Penetration into heat-killed onion epidermal cell layers (16 h.p.i). Fungal structures at the epidermal surface were stained with trypan blue (scale bar: 25 μm). F: Lesion formation on detached tomato leaves. Standard deviations are shown. G: Wettability test with water and 0.2% SDS on non-sporulating mycelia. Pictures were taken after 3 h. H: Wettability test with SDS solutions on sporulating aerial mycelia. Pictures were taken after 7 h.

The germination rates of hydrophobin knock-out mutants and the wild type strain were analysed under different conditions. As previously shown [13], wild type conidia incubated on glass without nutrients did not germinate to a significant extent, whereas nearly complete germination occurred in the presence of 10 mM fructose. On a hydrophobic polypropylene surface, conidia germinated to 90%. Neither the single nor the double nor the triple hydrophobin mutants showed any difference in their germination behaviour when compared to the wild type (Figure 3A). To test the viability of the conidia under long term storage conditions, they were incubated for up to 12 weeks at 20°C and 32% humidity in the dark. Samples were taken at regular intervals, and tested for germination of the conidia in full medium. No significant decrease in germination rates were observed for any of the mutant strains within this time period (data not shown), indicating that hydrophobin mutants of *B. cinerea *do not display obvious defects in conidial viability.

The mutants *Δbhp2*, *Δbhp3/bhp1 *and *Δbhp3/bhp2*, were also tested in a radial growth assay on TMA and Gamborg glucose agar, in the presence of high temperature stress (28°C on TMA), and under salt stress (0.5 M NaCl in Gamborg glucose agar). Again, no differences in growth rates of hydrophobin mutants and the wild type strain were observed (data not shown).

In *Verticillium dahliae*, the class II hydrophobin VdhI has been described to be required for microsclerotia formation [17]. The increased expression of *bhp2 *in sclerotia indicated that it could play a role in sclerotia formation or function. To induce sclerotia formation in the wild type strain and the hydrophobin mutants, conidial suspensions were inoculated on Gamborg glucose agar and incubated for 28 days in the dark. As shown for the hydrophobin triple mutant in Figure 3B, all of the hydrophobin mutants produced sclerotia in similar size and number as the wild type. When water droplets were applied to wild type and mutant sclerotia, they remained on the surface, indicating a hydrophobic nature of the sclerotial surface (not shown). The functional integrity of the sclerotia in the triple mutant and the *Δbhl1 *mutant was confirmed by a germination test (Figure 3C). Furthermore, microconidia and microconidia-forming structures were observed in close proximity to sclerotia in the wild type and in the mutants (Figure 3D; not shown for *Δbhl1 *mutant).

*Δmpg1 *mutants of *M. oryzae *are strongly impaired in their virulence on rice plants [4,18]. The *B. cinerea *hydrophobin mutants were therefore tested for host plant invasion and infection abilities. On onion epidermis cell layers, wild type strain B05.10 usually forms short germ tubes before penetrating into the epidermal layer. The hydrophobin mutants analysed in this test penetrated into epidermis cells with the same efficiency as the wild type (Figure 3E; not shown). For plant infection tests, one *Δbhp1*, one *Δbhp2*, one *Δbhp3*, three *Δbhl1*, three double and three transformants of the triple knock-out mutant were used to inoculate detached tomato leaves. No significant differences in the kinetics of lesion development and expansion were observed between any of the mutants and the wild type (Figure 3F, not shown). Similar infection tests performed with *Gerbera *and rose petals also did not reveal any phenotypic differences between the strains (not shown).

### Surface properties of conidia of hydrophobin mutants are indistinguishable from the wild type

In many fungi, deletion mutants lacking individual hydrophobins, especially of class I, show 'easily wettable' phenotypes, due to the reduction in surface hydrophobicity of mycelia and conidia. To test the *B. cinerea *hydrophobin mutants for a similar phenotype, they were inoculated onto rich nutrient media and grown for 12 days to obtain densely sporulating mycelium. Droplets of water and SDS solutions at different concentrations were carefully overlaid and incubated for up to 24 hours at 20°C in a humid chamber. As illustrated in Figure 3H, all of the droplets remained on the surface of sporulating mycelia of the wild type and the mutants. Even after 24 hours of incubation at high humidity, the droplets were still present, except that the droplets with 5, 10 and 18% SDS had partially sunken into the mycelia. Similarly, wettability tests performed on aerial hyphae of non-sporulating mycelia revealed no significant differences between the wild type and a hydrophobin triple mutant: Both strains were wetted by 0.2% SDS within a few minutes, while droplets of water remained on the mycelial surface for up to 7 hours (Figure 3G).

**Figure 4:**
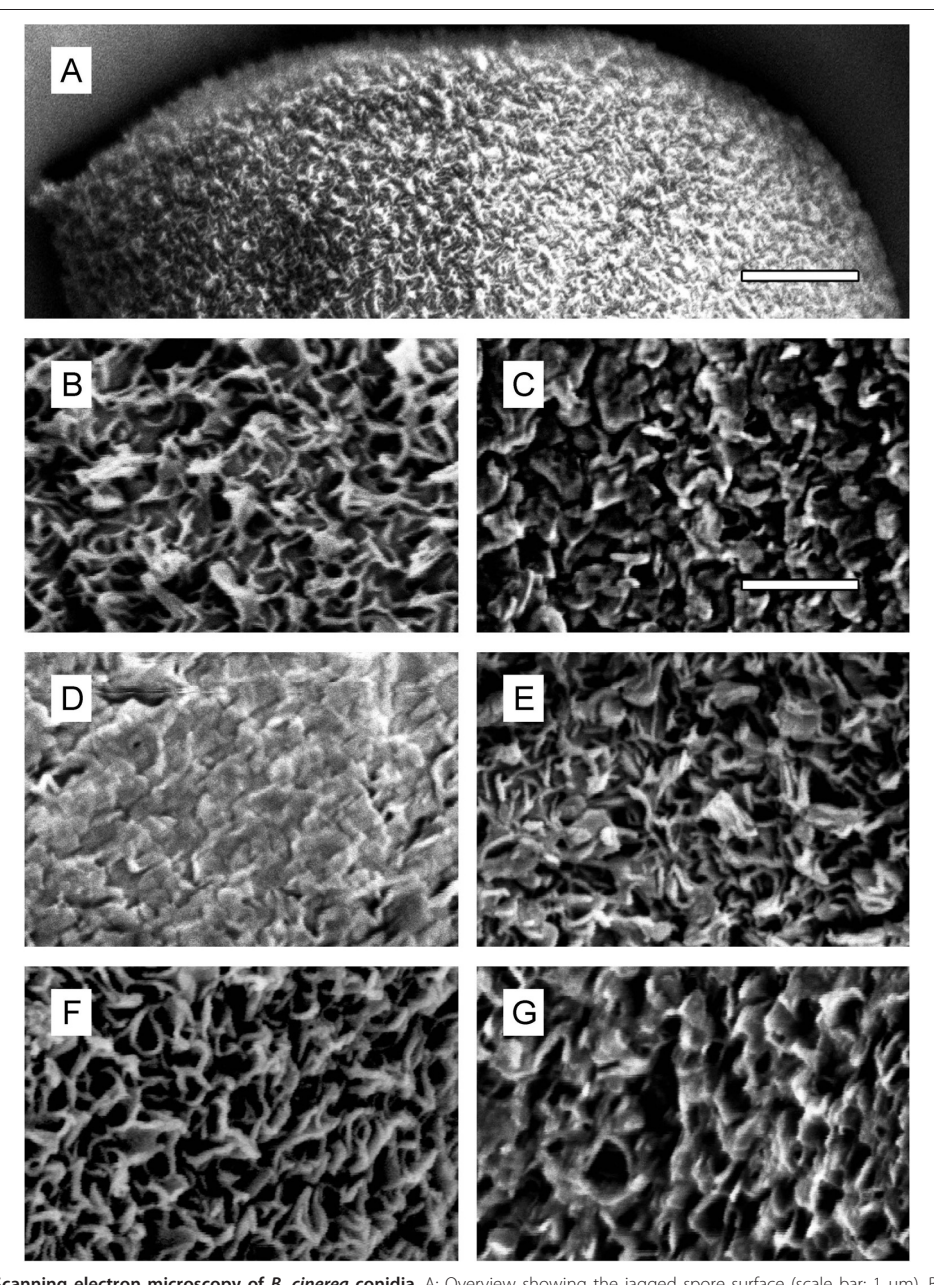
**Scanning electron microscopy of *B. cinerea *conidia**. A: Overview showing the jagged spore surface (scale bar: 1 μm). B, C: Higher magnifications, showing irregular jags of wild type (B) and triple mutant (C) spores. D: After treatment of wild type conidia with chloroform, the jags appeared abraded. E: Treatment of wild type conidia with hexane does not cause obvious changes in surface topography. F, G: Repeated washing with water caused minor abrasions of the spiny surface of wild type (F) and triple mutant (G) conidia. Scale bar for higher magnifications in B-G: 250 nm.

Conidia and hyphae of several fungi have been shown to be coated with hydrophobin layers that form typical rodlet-shaped crystalline structures. These layers are often absent in hydrophobin class I mutants [4,19-21]. Previous electron microscopy studies of *B. cinerea *conidia did not reveal evidence for rodlet-like surface structures [22]. To examine whether or not conidia of *B. cinerea *hydrophobin mutants were affected in surface morphology, scanning electron microscopy (SEM) with dryly harvested spores was performed. Neither the hydrophobin triple knock-out mutants nor the wild type conidia were covered with rodlet-shaped structures, and no differences were observed between the strains (Figure 4A-C). When wild type conidia were treated with hexane, only small changes in their surface structures were observed. Similarly, spores washed for several times with water left the conidial surface structures rather intact. In contrast, chloroform treatment had a drastic effect on the appearance of the conidial surface, leading to almost complete abrasion of the spinose surface (Figure 4D-G).

## Discussion

The genomes of filamentous basidiomycetes and ascomycetes generally contain multiple hydrophobin genes [2]. In contrast, hydrophobin genes have not been found in yeasts, for example *Cryptococcus neoformans*, *Saccharomyces cerevisiae*, *Schizosaccharomyces pombe*, and *Candida albicans*. Despite their important role, hydrophobins are not the only proteins that confer hydrophobic properties to fungal cell walls. The basidiomycete *Ustilago maydis *encodes a single hydrophobin, Hum2, and a much larger protein called Rep1. While Hum2 plays only a minor role, the peptides released from Rep1 during secretion are mainly responsible for conferring surface hydrophobicity to aerial hyphae in this fungus [23,24].

Our search in the annotated genome sequences of *B. cinerea *strains B05.10 and T4 has revealed the presence of three unambiguous hydrophobins, and a total of six hydrophobin-like proteins, according to the criteria defined in the results. For all except one of these genes, homologues in the closely related *Sclerotinia sclerotiorum *have been identified. In contrast, homologues in other fungi were only found for the three hydrophobins and for the hydrophobin-like protein BC1G_02483. BC1G_02483 was unusual because its size (234 amino acids), the dense spacing of the 8 consensus cysteines, and the presence of 4 additional N-terminal cysteines. The three hydrophobins share typical properties of class I (Bhp1) and class II (Bhp2, Bhp3) proteins. Expression of *bhp1*, *bhp2 *and *bhp3 *was found to be low in conidia and mycelium. This was confirmed by a qRT-PCR analysis that showed generally low expression levels of the three hydrophobin genes and the hydrophobin-like genes in conidia. However, Bhp1 was found to be strongly upregulated in fruiting bodies. This is supported by EST data from a cDNA library from apothecia of *B. cinerea*. Among 3189 ESTs, 15 (0.5%) were found to represent Bhp1 mRNA, while no ESTs of other hydrophobin sequences were identified in the apothecial library (J. Amselem and M.-H. Lebrun, personal communication). Our RT-PCR data did not provide evidence that deletion of the hydrophobin genes significantly changes the expression level of any other hydrophobin (-like) genes analysed in this study (Figure 2A; additional file 3: Figure S2).

Several of the hydrophobin (-like) protein encoding genes showed their highest expression levels either in sclerotia (*bhp2*, BC1G_12747) or in fruiting bodies (*bhp1*, *bhl1*). While we did not find any effects of the *Δbhp2 *mutants on sclerotia formation, the role of BC1G_12747 for sclerotia remains to be determined. Since we have not yet been able to perform crosses with *B. cinerea *in our laboratory, the role of Bhp1 and Bhl1 in fruiting body development and function also remains to be clarified. The strong upregulation of *bhp1 *and the apparently exclusive expression of *bhl1 *in fruiting bodies suggest that these genes might play a role during sexual development.

Using three different resistance markers for selection, mutants that lacked one, two, and all three hydrophobin genes *bhp1*, *bhp2 *and *bhp3 *were generated. To our knowledge, this is the first triple knock-out mutant described for *B. cinerea*. It was difficult to isolate because phleomycin is less suited for transformant selection compared to the commonly used hygromycin and nourseothricin, because of the growth of many false transformants. In addition to the hydrophobins, the hydrophobin-like gene *bhl1 *was knocked out. The resulting mutants were analysed for a variety of parameters of growth, differentiation and plant infection. In no case, significant differences between the phenotypes of wild type and mutant strains were observed. Specifically, the mutants showed wild type-like surface hydrophobicity of conidia and hyphae, and normal conidial surface structures when viewed by scanning electron microscopy. In agreement with a previous study [22], there is no evidence for the presence of a rodlet-like surface layer on *B. cinerea *conidia. This finding is in contrast to a variety of other fungi which have hydrophobin-coated cell walls surrounding conidia, germ tubes or aerial hyphae [2]. Interestingly, hydrophobin layers have been recently found to protect conidia from immune recognition [25]. While airborne conidia of *Botrytis *are usually less prevalent compared to the major genera *Cladosporium *and *Alternaria*, they have significant allergenic potential [26]. It is possible that this might be due to the absence of hydrophobin layers in *B. cinerea *conidia.

Our data indicate that *B. cinerea *hydrophobins do not play a major role in the hydrophobic coating of spores and hyphal wall, and thus are not important for attachment to hydrophobic surfaces or formation of aerial hyphae. Although we cannot completely exclude that any of the other five hydrophobin-like proteins listed in additional file 1: Table S1 are relevant in this respect, they are more hydrophilic than Bhp1, Bhp2, Bhp3 and Bhl1 and therefore not very likely to represent hydrophobins. As mentioned before, we do not exclude the possibility that Bhp1 or Bhl1 are involved in sexual development. Hydrophobins are known to be important for the formation of fruiting bodies in basidiomycetous mushrooms such as *Agaricus bisporus *and *Schizophyllum commune *[2]. In the chestnut blight fungus *Cryphonectria parasitica*, the class II hydrophobin cryparin has been shown to cover the walls of fruiting bodies and to be required for normal fruiting body development [27].

Because several hydrophobins are encoded in the genomes of filamentous fungi, it is difficult to fully assess their roles and to exclude complimentary functions. In the tomato pathogen *Cladosporium fulvum*, six hydrophobins have been identified. Using single mutations, one of them (Hcf1) was found to be required for spore surface hydrophobicity, another one (Hcf6) seems to be involved in adhesion of germinating spores to glass surfaces [28]. An attempt to assess the function of all hydrophobins simultaneously by multiple RNAi silencing failed to result in complete knock-down of the genes [29]. In *Fusarium verticillioides*, five hydrophobin genes (*hyd1*-*hyd5*) have been identified up to now in the genome. Phenotypical analysis of single mutants in these genes and of a *hyd1/hyd2 *double mutant revealed that *hyd1 *and *hyd2 *are required for normal microconidia formation, but did not provide evidence for a role of these hydrophobins in growth, infection behaviour, and mycelium hydrophobicity [16]. This indicates that in some fungi, including *B. cinerea *and *F. verticillioides*, hydrophobins play only a minor - if any - role in generating cell wall surface hydrophobicity. However, they might serve other, as yet unknown functions.

By far not all fungal spores contain superficial rodlet layers. For example, they are missing in the urediospores of rust fungi [30], and conidia of several powdery mildews [31]. Rust urediospores have been shown to be covered with a layer of lipids that can be extracted with organic solvents, leading to a significantly decreased hydrophobicity, and increased attachment to hydrophilic surfaces [32,33]. Surface bound lipids, containing hydrocarbon and fatty acid constituents, have been described for spores of several but not all fungal species analysed. The lack of visible effects of hexane treatment on the surface structure of *B. cinerea *conidia indicates that simple lipids are not a major surface component of these spores. Alternatively, proteins other than hydrophobins could play a role in conferring surface hydrophobicity. In *Stagonospora nodorum*, preformed surface glycoproteins have been proposed to play a role in the attachment of conidia to hydrophobic surfaces [34]. In the yeasts *S. cerevisiae *and *C. glabrata*, cell wall surface proteins called flocculins and adhesins, are involved in adhesion to various surfaces and in biofilm formation; their expression has also been correlated with an increased hydrophobicity of the cell surfaces [35,36]. Adhesin-like proteins are also encoded in the genomes of filamentous ascomycetes; however, their function remains to be analysed [37].

## Conclusions

Hydrophobins are very important for growth and differentiation of higher filamentous fungi, but their roles differ between different species. In some fungi, including *B. cinerea*, hydrophobic surface properties appear to be provided by as yet unknown mechanisms, different from the amphipathic layers formed by hydrophobins. It is evident that our knowledge about the molecules that cover the surfaces of fungal spores and determine their physicochemical properties is still far from being complete.

## Methods

### Cloning of the *B. cinerea bhp1*, *bhp2*, *bhp3 *and *bhl1 *genes and knock-out constructs

*B. cinerea *hydrophobin genes *bhp1*, *bhp2 *and *bhp3 *including flanking regions of 392-771 bp were amplified with primers (Table 2) BHP1-1/2, BHP2-1/2 and BHP3-1/2 (introducing *Bam*HI restriction sites at both ends of the PCR product) respectively from genomic DNA, and cloned into pBS(+) (Stratagene, La Jolla, USA). Subsequently, an inverse PCR was performed, using primers BHP1-3/4, BHP2-3/4 and BHP3-3/4. After digestion with *Eco*RI, the products were ligated with a hygromycin resistance cassette amplified by PCR from pLOB1 [38] with primers KO-Hyg1-EcoRI/KO-Hyg2-EcoRI, resulting in the plasmids pBHP1-Hyg, pBHP2-Hyg and pBHP3-Hyg. Knock-out constructs containing a nourseothricin resistance cassette were produced by replacing the hygromycin resistance cassette with a *Bam*HI/*Eco*RI restriction fragment from plasmid pNR2 [39,40], resulting in plasmids pBHP1-Nat and pBHP2-Nat. For the creation of hydrophobin triple mutants, a phleomycin resistance cassette from pAN8-1UM [41] was used. The *gpdA *promoter in pAN8-1UM was replaced by an *oliC *promoter fragment from pBHP1-Hyg using *Eco*RI/*Nco*I restriction sites. The modified phleomycin resistance cassette was amplified with primers T7/TtrpC-rev-EcoRV. The PCR product was digested with *Eco*RI/*Eco*RV and ligated with digested pBHP2-Hyg to replace the hygromycin resistance cassette, resulting in pBHP2-Phleo. For generation of the *bhl1 *knock-out construct, the gene was amplified with primers BHL1-1/2 (introducing *Bam*HI and *Xho*I sites), and cloned into pBSKS(+) (Stratagene). Inverse PCR was performed using primers BHL1-3/4 (introducing *Sma*I and *Hind*III sites), and the products ligated with the hygromycin resistance cassette cut out from pLOB1 using *Sma*I and *Hind*III, resulting in pBHL1-Hyg. Knock-out constructs for transformation were either amplified by PCR or cut out of the plasmid by digestion with *Bam*HI.

**Table 2:** Primers used in this study.

BcAct-RT-for	TCTGTCTTGGGTCTTGAGAG
BcAct-RT-rev	GGTGCAAGAGCAGTGATTTC

BcEF-RT1	ATGCTATCGACCCTCCTTCC

BcEF-RT2	GTTGAAACCGACGTTGTCAC

BHL1-1	CCGGGATCCGGGAATCTATCTGATAGCCAGTCAGTC

BHL1-2	GCACTCGAGGACGAGCTCTCCATGTCGTTTC

BHL1-3	ATACCCGGGACATGGTGTTGCTTGGTATGGTATGG

BHL1-4	TCGCAAGCTTTCATCTGGATGAAGCGGAGTCG

BHL1-Screen1	GCACAAGTATCTCGCTTCGGGTTC

BHP1-1	AAGGATCCACGTGGCAAAAGTGACTCTATCTA

BHP1-2	AAGGATCCATTTCTCAAGCTCTCCAAGTATC

BHP1-3	GAGAATTCTTTGAATATAGGGAGGAAGTCGTC

BHP1-4	GAGAATTCTGCCATTCCAATCGTTCTCTA

BHP1-Screen1	ACGAGTTATCAGCCGCGTAG

BHP2-1	AAGGATCCACGGGGCACATCACCATAGA

BHP2-2	AAGGATCCTGCTGCTCCGCAAAAGTCACA

BHP2-3	GAGAATTCGTTGTTTTCTTGAAGTTTGTTGTGA

BHP2-4	GAGAATTCGTTCTCCAGATAATTCATAGAGGAT

BHP2-Screen1	GGCCCTTCTAAGAGCACTAC

BHP2-Screen2	GCTGGGCTATATTGACCATC

BHP3-1	AAGGATCCTGCCCGCCATACATACACCT

BHP3-2	AAGGATCCAGCCACAGTCTCCCTCAATCA

BHP3-3	GAGAATTCAAGATGAGATGATGGATGAAGGAT

BHP3-4	GAGAATTCGCCGATTGTGATGGAAGTCTG

BHP3-Screen1	CGGACTTGGCACCTACTTAC

KO-Hyg1-EcoRI	GTGAATTCTGCAGCTGTGGAGCCGCATTC

KO-Hyg2-EcoRI	CTGAATTCCATGAATTGAAGCGGCACTGGC

OliC-inv	GATCGATTGTGATGTGATGGAG

Phleo-Screen	CGGAACGGCACTGGTCAACTTGG

T7	GTAATACGACTCACTATAGGGC

TtrpC-rev-EcoRV	GCCGATATCCGGCCGCTCTAGAAAGAAG

TubB-inv	AGTAGATGCCGACCGGGATC

01003-RT-for	CCTACCGCTCTAACAACAAC

01003-RT-rev	TTCCAACACCGGGCAATAC

01012-RT-for	CACAACCACCACACTTATCG

01012-RT-rev	TCCTTGAGCAGCACAGTATG

02483-RT-for	ACTTGTGCCTCGAATGATGG

02483-RT-rev	ATGAAGGAGTGACGGATTGG

03277-RT-for	TGTTGCGGAAGTCATCGAAG

03277-RT-rev	TCGGAATTCGTTGCGATTGG

03994-RT-for	TCAGCATGACTGCCCTATTC

03994-RT-rev	GAAGATCGCAGCACATGTTG

04521-RT-for	TGATGGGTTGGTTCCCTTTG

04521-RT-rev	GGGTTAGGATTGCAGCAGTATG

11117-RT-for	TTTGTGGCGGTAATGGCATC

11117-RT-rev	GTTCGTCCACAGTGGTTATC

12747-RT-for	TTCCTCACTCAAGCCCTCCTAAC

12747-RT-rev	ATCGGCATCGTAGAGCAATC

15273-RT-for	GTCGTTGCTATTCCCACTAC

15273-RT-rev	ATTTGCCTCCGAGCACGATAC

Introduced restriction sites are underlined.

### DNA and RNA preparation, cDNA synthesis and RT-PCR

Genomic DNA from *B. cinerea *strains was isolated as described [42]. Fungal RNA was purified, including a DNase treatment, using the NucleoSpin^® ^RNA Plant Kit (Macherey-Nagel, Düren, Germany). cDNA was synthesised with the Thermo Scientific Verso™ cDNA Synthesis Kit (ABgene House, Surrey, UK). For preparation of 15 hours old mycelium, 9 cm Petri dishes were inoculated with 2 × 10^6 ^conidia in 22.5 ml Gamborg medium (Duchefa, Haarlem, The Netherlands) with 10 mM KH_2_PO_4 _and 10 mM fructose, pH 5.5. The mycelium was harvested using a tissue cell scraper, and washed once with ice cold water before RNA preparation. Mature, fresh fruiting bodies from a laboratory cross of two *B. cinerea *field strains were harvested after 7 months, plunge frozen in liquid nitrogen, and lyophilized before RNA preparation (kindly provided by Sabine Fillinger, INRA AgroParisTech). RT-PCR was performed as described [43], using the primers 01003-RT-for/rev, 01012-RT-for/rev, 02483-RT-for/rev, 03277-RT-for/rev, 03994-RT-for/rev, 04521-RT-for/rev, 11117-RT-for/rev, 12747-RT-for/rev and 15273-RT-for/rev for detection of hydrophobin (-like) gene transcripts and BcEF-RT1/2 for amplification of an *ef1α *fragment as control (Table 2). Real-time RT-PCR was performed as described [44], using *actin *(primers BcAct-RT-for/rev) and *ef1α *as control. Expression of BC1G_04521 was not analysed by real-time RT-PCR, because of the multiple bands obtained by semiquantitative RT-PCR.

### Transformation of *B. cinerea *and screening of transformants

Two protocols were used for transformation of *B. cinerea*. Hydrophobin single and double knock-out mutants were produced according to the first method [45] and selected with 40 μg hygromycin B ml^-1 ^(Duchefa, Haarlem, The Netherlands) or 50 μg nourseothricin ml^-1 ^(Werner BioAgents, Jena, Germany) immediately added to the protoplasts in SH agar (0.6 M sucrose, 5 mM Tris-HCl pH 6.5, 1 mM (NH_4_)H_2_PO_4_, 0.8% bacto-agar). Generation of triple knock-outs was achieved with a second protocol as described [46], except that the complete transformation mixture was added to 200 ml of either SH agar (pH 7.3) or Czapek-Dox agar (pH 7.3, with 1 M sorbitol) containing 20 μg phleomycin ml^-1 ^(Zeocin™; InvivoGen, San Diego, USA). For selective growth of transformants, HA medium (1% [w/v] malt extract, 0.4% glucose [w/v], 0.4% yeast extract [w/v], pH 5.5, 1.5% agar) with 70 μg hygromycin B ml^-1 ^or 85 μg nourseothricin ml^-1 ^for hydrophobin single and double mutants, and Czapek-Dox agar (pH 7.3) with 50 μg phleomycin ml^-1 ^for triple knock-outs was used. Transformants were screened for homologous integration of knock-out constructs (primers for hygromycin resistance cassettes: BHP2-Screen1/TubB-inv, BHP3-Screen1/OliC-inv, BHL1-Screen1/TubB-inv; primers for nourseothricin resistance cassettes: BHP1-Screen1/OliC-inv, BHP2-Screen1/OliC-inv; primers for phleomycin resistance cassette: BHP2-Screen1/Phleo-Screen) and for the absence of wild type hydrophobin sequences (primers BHP1-1/2, BHP2-1/2 or BHP2-Screen1/BHP2-Screen2, BHP3-1/2, BHL1-Screen1/01003-RT-for; Table 2).

### Tests for germination, growth parameters and infection

Germination of conidia was tested on glass and on polypropylene surfaces in triplicates as described [13], either in water or with 10 mM fructose as a carbon source. Radial growth tests were performed once on TMA and Gamborg agar (0.305% [w/v] Gamborg B5 basal salt mixture [Duchefa, Haarlem, The Netherlands], 10 mM KH_2_PO_4_, 50 mM glucose, pH 5.5, 1.5% agar). The agar plates (9 cm diameter) were inoculated with 10 μl suspensions of 10^5 ^conidia ml^-1 ^in water, and incubated at 20°C in the dark for 3 days. TMA plates were also incubated at 28°C to induce heat stress. The differences in growth radius between days 2 and 3 were determined. Sclerotia formation of the mutants was tested twice on Gamborg agar [47], except that sclerotia were allowed to ripen for additional 14 days in the dark. Microconidia were collected from mycelium close to the sclerotia. The ability of mutants to penetrate into host tissue was determined once on heat-inactivated onion epidermis fragments. Infection tests were performed in triplicates on detached tomato leaves, and on gerbera and rose petals, as described previously [13]. To test sclerotia for germination, they were collected from six weeks old agar plates, rinsed for one minute in 70% [v/v] ethanol, and washed twice for 1 minute with sterile water. After transfer into Petri dishes filled with wet, sterile vermiculite, the sclerotia were frozen for 24 hours at -8.5°C and subsequently incubated at 20°C for one week under ambient light.

### Test for mycelium wettability

To obtain sporulating mycelium, HA and tomato malt agar plates were inoculated with a spore suspension and incubated for 12 days at ambient light. To produce non-sporulating mycelium, tomato malt agar plates were incubated for 4 days in a humid box in the dark. Aerial mycelia were overlaid with 20 μl droplets containing 50 mM EDTA and different concentrations of SDS [6], and incubated for up to 24 h in a humid box. Tests were performed in duplicates. Mycelia were evaluated as not wetted, if the droplets remained visible and were not absorbed by the aerial hyphae after the indicated incubation times.

### Scanning electron microscopy of *B. cinerea *conidia

Dry conidia from hydrophobin mutant strains were harvested from sporulating mycelium. For low-temperature scanning electron microscopy (LTSEM) spores were mounted on sticky sample holders and plunge-frozen in nitrogen slush. Samples were transferred into the Alto 2500 (Gatan, Oxford, UK) vacuum preparation chamber (pressure < 2 × 10^-4 ^Pa). Next they were sputter-coated with a 10 nm platinum layer prior to transfer on the SEM cryostage built into an S-4700 field emission scanning electron microscope (Hitachi, Tokyo, Japan). SEM micrographs were digitally recorded after samples were stabilised at 148 K at an acceleration voltage of 3 kV.

### Bioinformatic analyses

Nucleotide and amino acid sequences of the *B. cinerea *hydrophobins were taken from the databases of the Broad Institute (http://www.broadinstitute.org/annotation/genome/botrytis_cinerea.2/Home.html) and URGI (http://urgi.versailles.inra.fr/index.php/urgi/Species/Botrytis/Sequences-Databases). For amino acid sequence alignments the programs ClustalX 1.83 (ftp://ftp-igbmc.u-strasbg.fr/pub/ClustalX/) [48] and GeneDoc 2.5 (http://www.nrbsc.org/) [49] were used. Hydropathy plots were calculated with ProtScale (http://www.expasy.ch/cgi-bin/protscale.pl) [50] and drawn using Microsoft Excel. Prediction of signal sequences for secretion was performed using SignalP 3.0 (http://www.cbs.dtu.dk/services/SignalP/) [51,52]. GRAVY values were computed with ProtParam (http://www.expasy.ch/tools/protparam.html) [50].

## Authors' contributions

AM performed the experiments, except for scanning electron microscopy which was performed by KWM. ML co-supervised the project. AM and MH, who supervised the project, wrote the manuscript. All authors read and approved the final manuscript.

## Supplementary Material

Additional file 1 **Hydrophobins and hydrophobin-like proteins encoded in the genomes of *B. cinerea *and *S. sclerotiorum***. Click here for file

Additional file 2 **Hydropathy plots of Bhl1 in comparison to Mpg1 (A) and Mhp1 (B)**. Click here for file

Additional file 3 **RT-PCR-based expression analysis of hydrophobin genes in mutant strains *Δbhp1/bhp2*, *Δbhp3/bhp2 *and *Δbhl1***. Click here for file
