# Supplementary material for: Lack of evidence for a role of hydrophobins in conferring surface hydrophobicity to conidia and hyphae of Botrytis cinerea
Source: BMC Microbiol. 2011 Jan 13;11:10. doi: 10.1186/1471-2180-11-10 (PMC3032640; doi:10.1186/1471-2180-11-10)
Supplement: Additional file 1 — Hydrophobins and hydrophobin-like proteins encoded in the genomes of B. cinerea and S. sclerotiorum. [file 1471-2180-11-10-S1.PDF]

Table S1: Hydrophobins and hydrophobin-like proteins encoded in the genomes of *B. cinerea* and *S. sclerotiorum*.

B. cinerea		***S. sclerotiorum* strain 1980**		
Accession number B05.10 / T4 (name)	Size [aa]	Accession number	Size [aa]	Identity (Simil.) [%]
BC1G_15273 / BofuT4_P067050 (Bhp1) *	111*	SS1G_11895	111	84 (93)
BC1G_03994 / BofuT4_uP036020 (Bhp2) †	98†	SS1G_01214	98	63 (80)
BC1G_01012 / BofuT4_uP031610 (Bhp3)	98	SS1G_09248	94	49 (64)
BC1G_01003 / BofuT4_P031700 (Bhl1)	145	SS1G_09256 ‡	155‡	54 (66)
BC1G_02483 / BofuT4_P109960	234	SS1G_01590 ‡	230‡	79 (85)
BC1G_03277 / BofuT4_P101670	178	SS1G_06763	182	42 (62)
BC1G_04521 / BofuT4_P144500 §	167§ / 181§	SS1G_03897 §	127§	40 (49)
BC1G_11117 / BofuT4_P089080 †	109†	SS1G_01003	108	33 (50)
BC1G_12747 / BofuT4_P104570	106	No orthologue	-	-
(BC1G_04506) / no T4 orthologue #	(105)#	SS1G_08128 #	92#	67 (73)

Accession numbers are shown for *B. cinerea* strains B05.10 (Broad Institute website) and T4 (URGI website), and *S. sclerotiorum* 1980. Protein sizes including signal peptides, and similarities of *B. cinerea* und *S. sclerotiorum* bidirectional best hits (probably orthologues) are indicated. The low sequence identity between BC1G_04521 and SS1G_03897 is probably due to mis-annotations (see below). Homologues in other fungi were found only for the three hydophobins and BC1G_02483. *Mis-annotation of T4 sequence, manually corrected for comparison. †Probably mis-annotation of B05.10 sequence, manually corrected for comparison. ‡Probably mis-annotation of *S. sclerotiorum* sequence, manually corrected for comparison. §Mis-annotations of B05.10, T4 and 1980 sequences likely, which could not be corrected. #SS1G_08128 fits the criteria for hydrophobin-like proteins (see Results), while BC1G_04506 does not, because of the presence of two additional cysteine residues between the two cysteine doublets.

Details of manual sequence corrections: BofuT4_P067050: Coding region too long due to false intron annotation. BC1G_03994: Translational start is mis-annotated resulting in loss of N-terminal signal sequence; the correct annotation (BofuT4_uP036020) is available at URGI. SS1G_09256: Mis-annotations of exons and introns lead to false C-terminus, with no similarity to the *B. cinerea* sequences. SS1G_01590: Mis-annotation of start and stop codons, in addition sequence error manually corrected to allow alignment with *B. cinerea* homologue. BC1G_11117: Incomplete sequence, resulting in mis-annotation of start codon and loss of one cysteine residue, BofuT4_P089080 sequence is probably correct.
