# Supplementary material for: Lack of evidence for a role of hydrophobins in conferring surface hydrophobicity to conidia and hyphae of Botrytis cinerea
Source: BMC Microbiol. 2011 Jan 13;11:10. doi: 10.1186/1471-2180-11-10 (PMC3032640; doi:10.1186/1471-2180-11-10)
Supplement: Additional file 2 — Hydropathy plots of Bhl1 in comparison to Mpg1 (A) and Mhp1 (B). [file 1471-2180-11-10-S2.PDF]

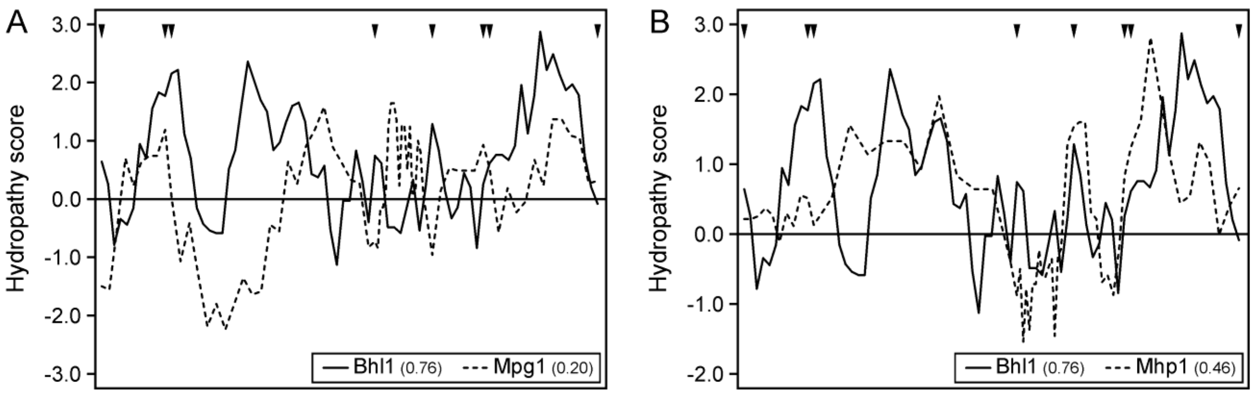

Figure S1: Hydropathy plots of Bhl1 in comparison to Mpg1 (A) and Mhp1 (B).

Hydropathy values were calculated for the region covering the eight cysteines (window size for calculation: 7 amino acids); positive values indicate regions of high hydrophobicity. Positions of cysteine residues are marked by triangles. Grand average of hydropathicity (GRAVY) of the analysed region is indicated in parentheses.
