# Supplementary material for: Lack of evidence for a role of hydrophobins in conferring surface hydrophobicity to conidia and hyphae of Botrytis cinerea
Source: BMC Microbiol. 2011 Jan 13;11:10. doi: 10.1186/1471-2180-11-10 (PMC3032640; doi:10.1186/1471-2180-11-10)
Supplement: Additional file 3 — RT-PCR-based expression analysis of hydrophobin genes in mutant strains Δbhp1/bhp2, Δbhp3/bhp2 and Δbhl1. [file 1471-2180-11-10-S3.PDF]

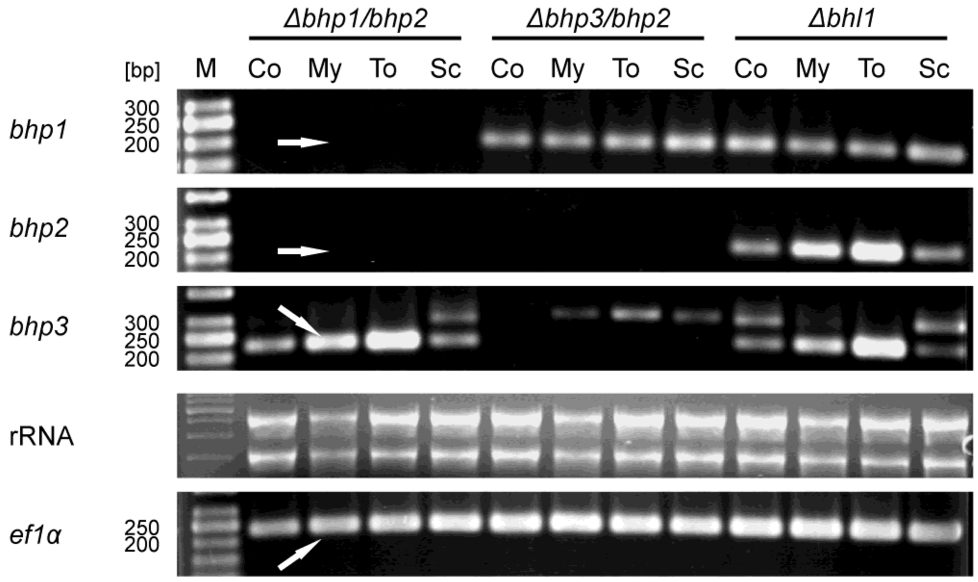

Figure S2: RT-PCR-based expression analysis of hydrophobin genes in mutant strains *Δbhp1/bhp2*, *Δbhp3/bhp2* and *Δbhl1*.

M: Size markers, with relevant sizes indicated; Co: Resting conidia; My: mycelium (15 h.p.i.); To: Infected tomato leaves (48 h.p.i.); Sc: Sclerotia. See Fig. 2 for water and genomic DNA controls. An EF1α encoding fragment was amplified as positive control. White arrows indicate positions of bands based on cDNA. Undiluted first-strand cDNA was amplified with 35 cycles, except for *ef1α* cDNA, which was amplified from 1:10 diluted first-strand cDNA. The PCR bands based on genomic DNA that were obtained for *Δbhp3* mutants in *bhp3*-specific reactions presumably result from a minor contamination of the knock-out strain with untransformed wild type nuclei.
